# Phage Lambda P Protein: Trans-Activation, Inhibition Phenotypes and their Suppression

**DOI:** 10.3390/v5020619

**Published:** 2013-02-06

**Authors:** Sidney Hayes, Craig Erker, Monique A. Horbay, Kristen Marciniuk, Wen Wang, Connie Hayes

**Affiliations:** Department of Microbiology and Immunology, College of Medicine, University of Saskatchewan, Saskatoon, S7N 5E5 Canada; E-Mails: sidney.hayes@usask.ca (S.H.); craigaerker@gmail.com (C.E.); mah134@mail.usask.ca (M.A.H.); kdm449@mail.usask.ca (K.M.); wew153@mail.usask.ca (W.W.); clh127@mail.usask.ca (C.H.)

**Keywords:** *E. coli* DnaB replicative helicase, bacteriophage lambda (λ) replication initiation protein P, allelic alterations of *dnaB* and P, ColE1 plasmid curing and replication inhibition, cellular filamentation

## Abstract

The initiation of bacteriophage λ replication depends upon interactions between the *ori*λ DNA site, phage proteins O and P, and *E. coli* host replication proteins. P exhibits a high affinity for DnaB, the major replicative helicase for unwinding double stranded DNA. The concept of P-lethality relates to the hypothesis that P can sequester DnaB and in turn prevent cellular replication initiation from *oriC*. Alternatively, it was suggested that P-lethality does not involve an interaction between P and DnaB, but is targeted to DnaA. P-lethality is assessed by examining host cells for transformation by ColE1-type plasmids that can express P, and the absence of transformants is attributed to a lethal effect of P expression. The plasmid we employed enabled conditional expression of *P*, where under permissive conditions, cells were efficiently transformed. We observed that ColE1 replication and plasmid establishment upon transformation is extremely sensitive to P, and distinguish this effect from P-lethality directed to cells. We show that alleles of *dnaB* protect the variant cells from *P* expression. P-dependent cellular filamentation arose in *ΔrecA* or *lexA*[Ind^-^] cells, defective for SOS induction. Replication propagation and restart could represent additional targets for P interference of *E. coli* replication, beyond the *oriC*-dependent initiation step.

## 1. Introduction

**Figure 1:**
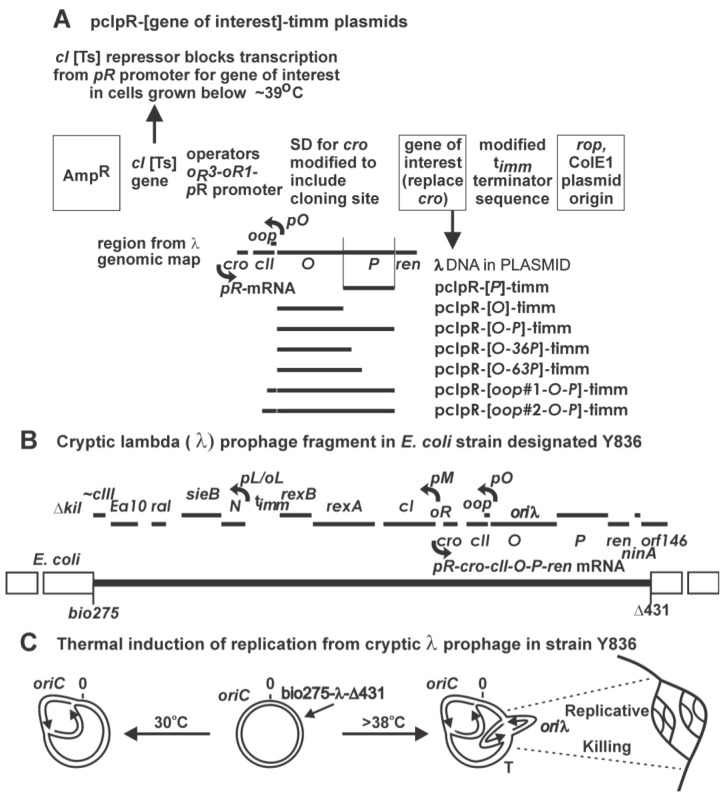
Relevant λ genes in expression plasmids and prophage. (**A**) Arrangement of synthetic plasmid pcIpR-[GOI]-timm. Plasmids employed include the precise coding sequence for GOI (gene of interest) genes *O, P,* or alleles of *P*, including *P*^π^, *P*-SPA, *P*^Δ76^, each inserted, in precisely the same orientation and position as gene *cro* in λ, directly downstream of *pR*. The coding sequence for the GOI is terminated by an ochre stop codon inserted just ahead of the powerful *timm* termination sequence [16,17], previously named *ti*[18,19]. The regulatory regions of pcIpR-(GOI)-timm were described (Figure 1 plus supplemental sequence file in [20]). The expression of the GOI from promoter *pR* is negatively regulated by the lambda CI[Ts] repressor binding to the wild type *oR* operator sites overlapping *pR*. These plasmids do not contain the *oL* operator sequences so that the tightest repression of transcription from the *pR* promoter, requiring CI-mediated DNA looping via CI dimers binding both operator sites [21], is not possible. Transcriptional read through beyond the GOI is prevented by *timm*, shown in its natural map position between the left operators, *oL* and the C-terminal end of *rexB* (see gene map drawn in Figure 1B). The synthetic sequence from the translational termination sequence for GOI through *timm* to downstream *EcoR*I-*Sal*I is: *TAATCGAT*ccc*ggGG*tcagc*C*ccgggttttcttt*TGAATTCGTCGAC*, where the bases that were modified from the wild type lambda DNA sequence are in capitalized italics. Shifting cells with a pcIpR-[GOI]-timm plasmid that were grown at 25 or 30 ˚C, to above 39 ˚C induces expression of the GOI from the plasmid [20]. (**B**) Cryptic prophage in strain Y836. The λ phage genes *cro-cII-O-P-ren* are transcribed from promoter *pR* that is embedded within the rightward operator sequence, *oR*, between genes *cI* and *cro*. Gene *cI*, encoding a temperature sensitive repressor is transcribed from promoter *pM*; and *cro* is transcribed in the opposite direction from *pR*. C. At temperatures where CI remains active, i.e., at or below 38 ˚C, λ replication initiation is prevented, and the λ fragment is replicated as part of the *E. coli* chromosome by forks arising from *oriC*. At about 39 ˚C, CI becomes fully denatured, *pR* transcription is induced, and λ undergoes a few replication initiation events from *ori*λ [22].

The mechanism for bi-directional initiation of bacteriophage λ DNA replication involves a complex interaction of λ proteins O and P with the *E. coli* host DNA replication machinery. The arrangement of genes *O* and *P* on the λ genome is shown in Figure 1. Their expression depends upon *pR-cro-cII-O-P* mRNA synthesis from promoter *pR* which is negatively regulated by the CI repressor, made from gene *cI*, binding to *oR* operator sequences overlapping *pR*. The gp*O* (= O) acts to bind *ori*λ, the λ origin of replication, situated midway within the *O* sequence [1]. The gp*P* = P protein facilitates replication initiation through recruitment of an *E. coli* host protein(s) to form a preprimosomal initiation complex at *ori*λ. In agreement with earlier genetic studies, P was found to physically interact [2] with DnaB the major replicative helicase for unwinding double-stranded (ds) DNA. DnaB promotes the advancement of a growing replication fork [3] by using energy provided by ATP hydrolysis; it functions as a “mobile promoter” in the general priming reaction [4,5] to aid DnaG primase in producing RNA primers for extension by DNA polymerase III; and it is able to promote progression of Holliday Junctions in the repair of DNA damage [6,7]. DnaB is a hexameric homomultimer with up to six bound ATP molecules [8,9]. Its intrinsic ssDNA binding activity is inhibited when the free form of DnaB forms a complex with the host replication initiation protein DnaC ([10] and included references). P can commandeer DnaB away from DnaC [11]. P:DnaB participates in loading DnaB onto DNA during formation of the DnaB:P:O:*ori*λ preprimosomal complex. The interaction between P and DnaB inactivates the helicase activity of DnaB. Restoration of DnaB activity, coupled with activation of *ori*λ-dependent replication initiation involves the dissociation of P bound to DnaB by *E. coli* heat shock proteins DnaK, DnaJ, and GrpE [12,13]. The ability of P to dissociate preformed cellular DnaB-DnaC complexes, coupled with a low cellular concentration of DnaB (~20 hexamers/cell) [14,15], suggests the hypothesis that λ *P* expression can inhibit *E. coli* DNA replication initiation from *oriC*, alluded to herein as P-lethality.

Klinkert and Klein [23] examined the effect of cloning the λ fragment sequence bp’s 39168 – 44972 (part of *O*, *P-ren-*and downstream* orf’s 290-146-57-56-60-204-68-64*) into a plasmid expression vector regulated by the *lac* promoter. They showed that inducing *P* expression correlated with effects of shifting cells with temperature sensitive slow-stop mutations in replication initiation genes to the non-permissive temperature, i.e., an eventual inhibition in *E. coli* replication was seen after about a 50 min induction. The effect paralleled the addition of chloramphenicol, and was opposite the effect of adding nalidixic acid, which produced an immediate (fast-stop) inhibition in chromosomal replication (their Figure 5). Maiti *et al.* [24] cloned λ genes into pBR322 and followed the survival of bacteria after transformation with a plasmid that constitutively expressed *pR-cro-O-P-ren-tR2*, measuring transformants (Amp^R^ colony forming units, cfu) per μg DNA. They observed that plasmids expressing active *P* did not yield survivor cfu, concluding that *P* expression is lethal to the host, i.e., P-lethality. They found that three types of *groP* mutants of *E.coli*, which do not allow λ DNA replication, possibly due to the lack of interaction of P protein with the altered DnaB (*gro*PA15, *gro*PB558) or DnaJ protein (*gro*PC259), were equally susceptible to killing by P protein. They concluded that P-lethality *does not involve interaction of* P with the host DnaB, DnaJ, or DnaK proteins, which are all essential for λ DNA replication [25,26,27] but is targeted to the *E. coli* DNA replication initiation protein DnaA, inhibiting its *oriC* DNA binding [28]. Datta *et al.* [29] identified mutations within the gene *dnaA* that confer cellular resistance to P-lethality. An alternative hypothesis for the absence of transformants formed by plasmids expressing *P* was not considered in prior studies, i.e., that *P* expression from the transformed pBR322-derived plasmids employed interfered with / blocked the initiation of plasmid replication and copy increase within the newly transformed cell. Since the replication origin of pBR322 was derived from pMB1, closely related to ColE1 [30] we refer herein to the plasmids employed as ColE1-type plasmids.

In the present manuscript we examine if the interaction of P and DnaB provides an alternative mechanism for P-lethality. We assess the influence of P expression upon the maintenance of ColE1 plasmids, and we examine whether P expression, shown to stimulate cellular filamentation, depends upon triggering a cellular SOS response. 

## 2. Result and Discussions

### 2.1. Transformation of Cells by Plasmids Expressing P, Complementation and Immunity Assays

Plasmid pHB30 (Table 10), encoding an intact lambda *P-ren* sequence whose expression is regulated by the CI[Ts] repressor, yielded similar transformation results as those reported by Maiti *et al.* [24], i.e. no transformants were observed at 42 ˚C for *dnaB*^+^ hosts, or for several host strains isolated as being defective for λ replication, i.e., *groP*A15, *groP*558, *grpE*280, *grpC*2 [31]. However two mutants employed in this study, *grpA*80 and *grpD*55 [32] supported pHB30 transformation at 42 ˚C at about the same efficiency as at 30 ˚C [31]. The *grpD*55 mutation (initial reported *E. coli* linkage-map position ~71.5 min) was re-mapped as an allele of *dnaB* and was co-transduceable withmalF3089::Tn*10* at ~91.5 min [33]. (We were unable to move the grpA80 mutation into another host.) These results were considered preliminary because pHB30 included several genes (a *cro-O* gene fusion, *P* and *ren*), and because the *grpA80* and *grpD55* mutations were not then confirmed to fall within *dnaB*. Table 1 shows that both *grpA*80 and *grpD*55 mutations each possess two missense changes within *dnaB*, one being in common, E426K.

**Table 1:** Sequencing analysis of alleles of *dnaB*.

*dnaB* allele	bp *E. coli* mutation	AA changed
*grpD55*	4,263,102 G to A	V256I
	4,263,612 G to A	E426K
*grpA80*	4,263,349 G to A	G338E
	4,263,612 G to A	E426K

The ability of the *dnaB-grpD55* allele within *E. coli* strain 594 to inhibit λ vegetative growth is shown in Table 2.

**Table 2:** Inhibition of λ*cI*72 vegetative growth on *dnaB-grpD55* host.

Host strains	EOP on host cells incubated at temperature^ a^			
	30	37	39	42 ˚C
594	1.0	nd^b^	nd	0.94
594 *grpD55*	0.0147	4.4 × 10^-7^	4.4 × 10^-7^	4.6 × 10^-7^

^a^: Efficiency of plating (EOP) of λ*cI*72 was determined by dividing the average phage titer on 594 *dnaB-grpD55* host cells by the titer on 594 host at 30 ˚C (= 6.8 × 10^9^ pfu/ml). ^b^: nd = not determined.

Plasmid pcIpR-*P*-timm (Figure 1A) includes λ gene *cI*[Ts]857, encoding a thermolabile repressor that is expressed from promoter *pM*, and a precise copy of wild type gene *P* DNA, situated downstream from λ promoter *pR* in exactly the same configuration as is occupied by gene *cro* in the λ genome. The expression of *P* from this plasmid is regulated by the CI[Ts] repressor binding to the *oR* sequences overlapping *pR*. The expression of *P* from pcIpR-*P*-timm was initially assumed similar to that shown for D-CAP or D-SPA gene fusions, as in pcIpR-D-CAP-timm (Figure 4 in [20]), where there was no detected expression from the non-induced gene fusion. The expression of genes *P* and *cI*[Ts]857 from pcIpR*-P-*timm was assessed by complementation, measuring the biological activity of P, and a CI-immunity assay to assess CI[Ts] repression of *imm*λ phage plating. The complementation assay determined whether P expressed from the plasmid could support the plating of a phage with an amber mutation in *P*, i.e., the heteroimmune infecting phage λ*imm*434*P*am3, which is insensitive to repression by CI^λ^. The ability of cells with pcIpR-*P-*timm to produce CI[Ts] at a sufficient level to turn off *pR* transcription from both plasmid or an infecting phage was assessed by its inhibition of λ*cI*72 plating.

Table 3 shows the transformation of nonlysogenic 594 *dnaB*^+^ cells (*dnaB* was sequenced [34]) and of 594(λnin5) lysogenic cells. The CI repressor made from the λnin5 prophage is not Ts. The lysogenic cells expressing CI^+^ were transformable by pcIpR-*P*-timm at 37 ˚C. The nonlysogenic cells were transformed by pcIpR-*P*-timm only between 25-34 ˚C, where CI[Ts] retains sufficient repressor activity to shut off *P* expression in transformed cells. CI[Ts] is reported [35] marginally active at ≥37 ˚C and would not fully repress *P* expression, explaining the absence of transformants arising in nonlysogenic cells when the transformation temperature is raised above a temperature where CI[Ts] repressor retains sufficient DNA binding activity.

**Table 3:** Cellular transformation by pcIpR-*P*-timm plasmid.

Cells Transformed	Plating Temp. ˚C	Transformation Frequency ^a^	Transformants per µg DNA
594	25	5.2 × 10^-5^	1.5 × 10^5^
	37	<1.7 × 10^-8^	0
594(λ wt nin5)	25	7.2 × 10^-5^	4.7 × 10^4^
	37	8.5 × 10^-6^	5.5 × 10^3^

^a^: Transformation frequency represents the average cell titer on TB+Amp_50_ plates per average cell titer on TB plates.

The 594[pcIpR-*P*-timm] cells complement for P, at 30 ˚C, and very marginally (in comparison to the Δ*P* plasmid) complement at 25 ˚C (Table 4), suggesting a low level of leaky *P* expression, even when CI[Ts] is active.

**Table 4:** Complementation of λ *P*am mutation by plasmid cloned *P* gene.

Plating host	Incubation temperature	EOP ^a^ of λ*imm*434*P*am3
594[pcIpR- *P*-timm]	25 ˚C	0.0008
	30	0.82
	37	0.93
594[pcIpR- *P*^Δ76^-timm]	30	<0.00003
	37	2 × 10^-7^

^a^: EOP of 1.0 determined on host TC600 *supE* at 30 ˚C.

The noninduced P-SPA fusion complements for P in cells grown at 30 and 37 ˚C (data not shown). 594[pcIpR-*P*^Δ76^-timm] transformants were unable to complement for *P*, suggesting that the region of *P* removed by the in-frame deletion of 76 codons, localized to the N-teminal end of the 233 amino acid P protein (fusing part of codon 9 with 86) is essential for the role of P in λ replication. The immune state, i.e., *imm*λ[Ts] phenotype, of the pcIpR-[ ]-timm plasmids was examined (Table 5, and [36]) by plating λ*cI*72 on hosts with plasmids encoding intact *P* or *O* genes.

**Table 5:** EOP ^a^ of λ*cI*72 on host strains with *imm*λ[TS] or hybrid *imm* plasmids ^b^

Host strains	Plating temperature (˚C)					
	25	30	35	37	39	42
594	0.5	0.9	0.9	1.0	1.0	0.4
594[pcIpR-*P*-timm]	<4 × 10^-10^	<4 × 10^-10^	0.2	0.5	0.7	1.0
594[pcIpR-*O*-timm]	<4 × 10^-10^	<4 × 10^-10^	<4 × 10^-10^	9 × 10^-7^^c^	2 × 10^-6^^c^	0.8
594[p434’pR-*O*-timm]	0.8	0.9	1.0	1.0	1.0	1.0

^a^: EOP (efficiency of plating) of 1.0 determined for λ*imm*λ*cI*72 on host 594 at 37 ˚C. ^b^: The CI[Ts] repressor made from the pcIpR plasmids blocks the vegetative growth of an infecting *imm*λ phage as λ*cI*72, at or below ~39 ˚C, as well as the expression of genes *O* or *P*, inserted downstream of the *pR* promoter. Plasmid p434’pR-*O*-timm has a hybrid *cI* gene fusion made from λ and 434 *cI* genes and the resulting repressor does not repress transcription from *pR*, allowing constitutive *O* expression at all temperatures [36]. ^c^: Rare min plaques can only be seen under stereo microscope.

**Figure 2:**
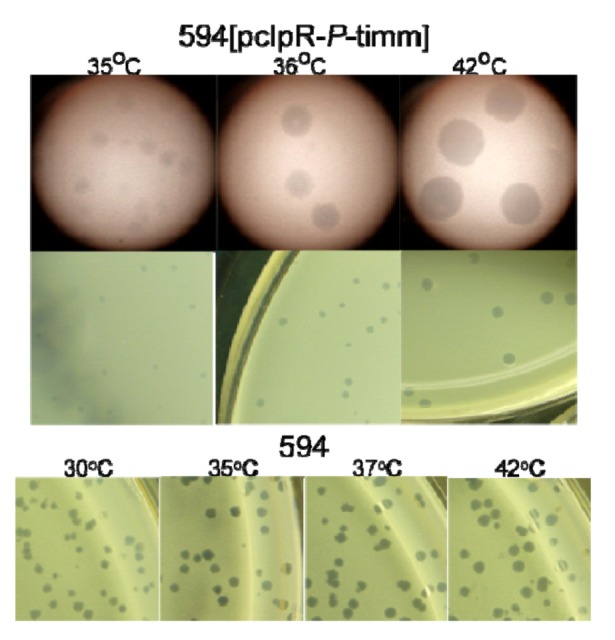
Plaque type / formation by λ*cI*72 on 594[pcIpR-*P*-timm] and 594 host cells. The ability of λ*cI*72 to form plaques on 594[pcIpR-*P-*timm] host cells at 35-42 ˚C is attributed to loss, or considerable reduction, in plasmid copy number per cell. The *imm*λ interference phenotype (compare EOP, Table 5, 25 and 30 ˚C *vs.* 35 ˚C) is dependent upon CI[Ts] repressor expressed from the plasmid. The cellular loss of *imm*λ interference correlates with plasmid loss and the observed increase in plaque size between 35 and 42 ˚C; whereas, the plaque size was essentially constant between 30 to 42 ˚C when λ*cI*72 was plated on 594 cells without the plasmid. The photos of the individual plaques shown in the top row were taken through the lens of a stereo microscope, from agar overlay plates (middle row) incubated at the indicated temperatures.

The plasmid control p434’pR-*O*-timm with a hybrid λ/434 CI repressor does not block *pR* transcription, expresses *O* constitutively at all temperatures, and showed no immunity to an infecting *imm*λ phage. Plasmid pcIpR-*O*-timm confers high *imm*λ immunity in cells grown between 25 and 39 ˚C, and loses it at 42 ˚C where the CI[Ts]857 repressor is fully inactive. Additional immune characterization of these plasmids was reported (Table 4 in [36]). Although pcIpR-*O*-timm only carries *oR* operators for repressor binding, CI immune repression is maintained through 39 ˚C, but lost at 42 ˚C where the CI repressor is denatured. Thus, the pcIpR-(GOI)-timm plasmids, even though they carry only *oR* site, can maintain CI immune repression, and presumably block the expression of GOI positioned downstream of *pR*, between 25-39 ˚C. However, the analogous plasmid pcIpR-*P*-timm exhibits *imm*λ immunity at 25-30 ˚C but the immune response is ineffective at and above 35 ˚C. Phage λ*cI*72 forms tiny to small plaques on 594[pcIpR-*P*-timm] cells at 35 and 36 ˚C where the CI[Ts]857 repressor is somewhat active [35], and larger plaques at 42 ˚C where the repressor is inactive (Figure 2).

#### 2.1.1. “Trans-activation” of P

The gene *P* function of an *imm*λ prophage was previously shown to be *trans*-activatable [37,38], i.e. cells with a repressed λ prophage could fully complement and support the vegetative growth of an infecting *imm*434 phage defective for *P* (by a factor of 10^3^), even though the expression of the prophage copy of *P* was repressed by the *imm*λ CI repressor blocking *pR* transcription in prophage with both *oL* and *oR* sites. Thomas [38] argued that “a small but significant amount of P product was synthesized by the prophage”, which he rationalized as being explained by the existence of a minor constitutive promoter mapping between *pR* and *P* on the prophage genome. We saw *trans*-activation of *P* from repressed pcIpR-*P*-timm (slightly at 25 ˚C and nearly full complementation at 30 ˚C), which is explained by leaky transcription from *pR*, since there is no room for an unaccounted promoter upstream of *P* as only 18 bases, including the ribosomal binding site, separates the start site for *pR* mRNA transcription and the AUG for *P*. We conclude that trace, certainly not full, *P* expression is sufficient for *ori*λ dependent replication initiation.

### 2.2. Influence of P-Expression on Plasmid Retention.

**Figure 3:**
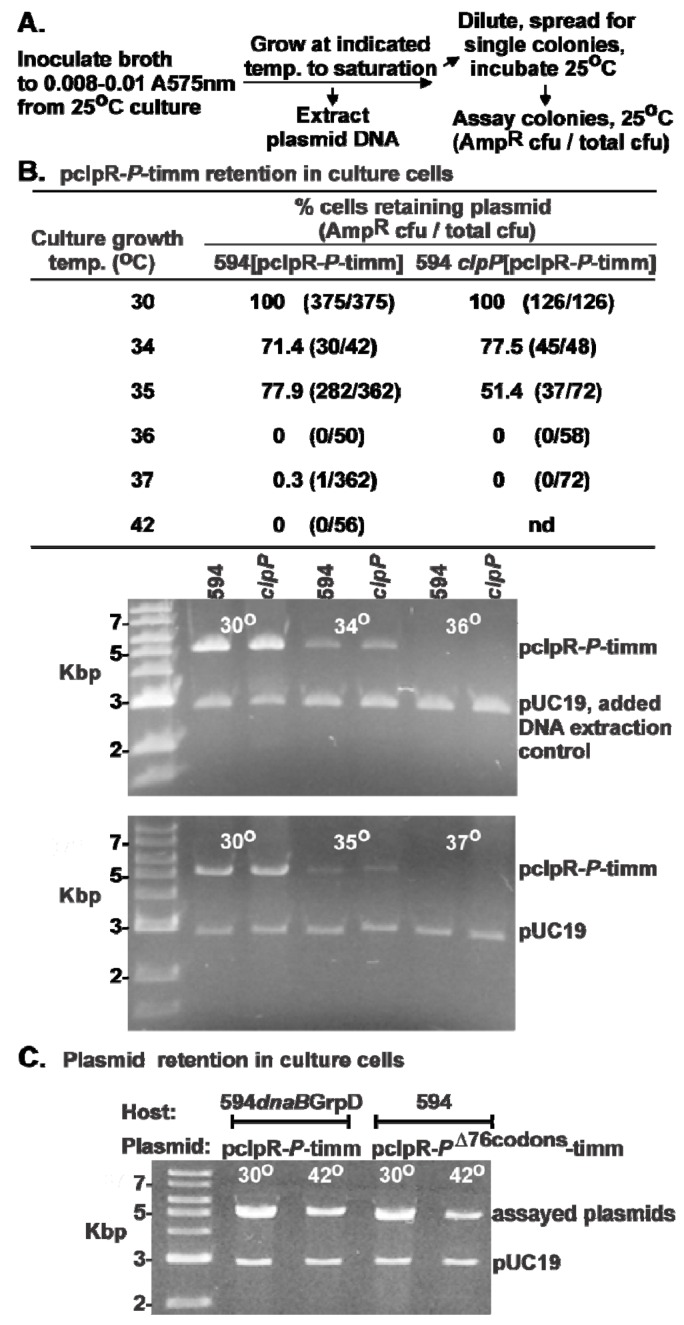
*P*-induced plasmid loss. (**A**) Cultures of 594[pcIpR-*P*-timm], 594 *clpP*::kan[pcIpR-*P*-timm], 594 *grpD55*[pcIpR-*P*-timm], and 594[pcIpR-*P*Δ76-timm] were grown to stationary phase in TB plus 50 μg/ml ampicillin for 48 hr at 25 ˚C. Cell aliquots were diluted into fresh TB medium (no ampicillin) as shown in the outline (A) and incubated for about 20 hr in shaking bath between 30 to 42 ˚C (refer to Experimental Section *3.8*). (**B**), (**C**) Plasmid retention by culture cells (described in (A)) grown between 30 to 42 ˚C.

The *in*ability of plasmids expressing *P* to form stable transformants is used as a measure of “P-lethality”, based on an assumption that P made from the transforming plasmid kills the transformed cells. The influence of *P* expression from pcIpR*-P-*timm in 594 *dnaB*^+^ cells was examined for plasmid replication / maintenance / stability (Figure 3). Plasmid loss was observed in cultures of 594[pcIpR*-P-*timm] grown at 34 or 35 ˚C (in comparison to cultures grown at 30 ˚C), but curing was extensive in cell cultures grown at or above 36 ˚C (Figure 3B). Essentially identical results were seen for 594 *clpP*[pcIpR-*P*-timm] cultures (Figure 3B) showing that inactivation of the *clpP* protease, which participates along with ClpX in degrading λ O protein [39,40], did not significantly influence P-dependent plasmid loss relative to the *clpP*^+^ strain 594; the ClpP protease defect did not amplify plasmid loss, which is expected if its absence increased the P concentration per cell. Secondary cultures prepared from 594 *grpD55*[pcIpR*-P-*timm], or 594[*pcIpR-P*^Δ76^*-*timm] cells retained their plasmids, even when grown at 42 ˚C for at least seven doublings while constitutively expressing *P* (Figure 3C). The *grpD55* allele blocks λ vegetative growth (Table 2) and permits cell survival (at some level) in the presence of constitutive *P* expression at 42 ˚C, suggesting that allelic variations in DnaB *can* protect cells from *P* expression and its consequent negative influence on cell metabolism. This result is in disagreement with the hypothesis made by Maiti *et al.* [24], which in essence states that allelic variants of *dnaB* will not protect cells from P-lethality.

### 2.3. P-Lethality Suppression

The results in Figure 3 do not account for P-lethality, only plasmid loss, and so their interconnection was examined (Table 6). Whereas cells within a starting culture that become cured of a plasmid expressing P can grow and eventually fully populate a culture, isolating individual cells by spreading dilutions on an agar plate will permit determination of cell viability at the incubation temperature. Less than 1% viability was observed for spread 594[pcIpR*-P-*timm] cells incubated at or above 37 ˚C, and any surviving cfu had lost the plasmid. *P* expression was not lethal in spread 594 *grpD55*[pcIpR*-P-*timm] cells incubated between 37 and 42 ˚C, where the cfu arising at or above 37 ˚C retained the plasmid. We examined whether the plasmids extracted from these survivor cfu retained P-lethality and plasmid-loss properties, when moved back into 594 *dnaB*^+^ cells. The reclaimed plasmids retained both the P-lethality and plasmid loss phenotypes (bottom lines, Table 6).

**Table 6:** Influence of modifying *P* or *dnaB* on P lethality and plasmid loss.

Host cells and plasmids	Cell viability^ a^ and (plasmid retention/cfu; %) ^b^ at growth temperature (˚C)		
	25	Ave 37 & 39	42
594[pcIpR- *P*-timm]	1.0	0.0049	0.001
	(195/196; ~100)	(0/57; 0)	(0/28; 0)
594[pcIpR- *P*^SPA^-timm]	1.0	0.006	0.004
	(14/14; 100)	(0/150; 0)	(0/39; 0)
594[pcIpR- *P*^π^-timm]	1.0	0.635	0.003
	(103/103; 100)	(789/793; ~100)	(0/294; 0)
594[pcIpR- *P*^Δ76^-timm]	1.0	0.96	0.12
	(166/168; 99)	(334/336; 99)	(166/168; 99)
594 *grpD55* [pcIpR-*P*-timm]	1.0	0.99	1.0
	(35/35; 100)	(87/88; 99)	(14/14; 100)
594 *grpD55* [pcIpR-*P*^SPA^-timm]	1.0	0.75	0.57
	(42/42; 100)	(88/94; 94)	(18/19; 95)
594 *grpD55* [pcIpR-*P*^π^-timm]	1.0	0.08	0.01
	(54/54; 100)	(434/435; ~100)	(5/108; 5)
594 *grpD55* [pcIpR-*P*^Δ76^-timm]	1.0 (nd)	0.45 (nd)	0.001 (nd)
Re-claim pcIpR-*P*-timm from 594 *grpD55* cultures and transform into 594 *dnaB*^+^ cells			
Re-claim from 25˚C cultures ^c^	1.0	0.08	0.01
	(54/56; 96)	(0/119; 0)	(0/91; 0)
Re-claim from 42˚C cultures^ d^	1.0	0.059	0.026
	(130/130; 96)	(1/230; 0.4)	(0/201; 0)

^a ^: The cell viability shown in each column entry, in top line, was determined by dividing the cell titer obtained at each given incubation temperature by the cell titer at 25 ˚C.) Refer to Experimental Section *3.5.* ^b^: The values in parentheses in each column entry show the number of Amp^R^ cfu / number of survivor cfu assayed per indicated temperature; the value following represents the percentage of Amp^R^ cfu with plasmids, and was rounded up. All data show the results for two or more independently transformed single colonies. ^c ^: Exp.’s for sc1, sc2. ^d ^: Exp.’s for sc’s 3,4,5, 6.

A stark contrast was noted between the *O* and *P* versions of pcIpR-timm plasmids regarding their ability to prevent the plating of *imm*λ phage λ*cI*72. The *O*-version retained the *imm*λ phenotype between 25 and 39 ˚C, losing it when the CI[Ts] repressor became denatured; the *P*-version lost it between 30 and 35 ˚C and supported plaque formation on the cell lawns at 35 and 36 ˚C. The simplest explanation is that the *P*-version plasmid was lost from the cells grown above 30 ˚C, and coordinately, *cI* repressor gene expression was lost. Gel analysis showed that 100% of cfu from cultures grown at 30 ˚C retained the *P*-version plasmid, but cultures grown at 34 and 35 ˚C were reduced in plasmid copies, and cultures grown at or above 36 ˚C were fully cured. Since cultures grown at 30 ˚C could complement for *P*, plasmid maintenance was very sensitive to trace *P* expression, which apparently increased with an increase in culture temperature from 30 to 36 ˚C. In support of this claim, we obtained 1.5 × 10^5^ transformants per μg of pcIpR-*P*-timm DNA at 25 ˚C and none at 37 ˚C. The partial to full loss between 34 – 37 ˚C (Figure 3) of *P*-encoded plasmids, argues for trace levels of P causing ColE1 plasmid curing. This result suggests a new dimension to the concept of P-lethality. It appears that ColE1 plasmid replication is extremely sensitive to P^λ^ protein and that P-inhibition of plasmid replication prevents plasmid establishment upon transformation. This provides a better explanation for the lack of transformants (found by us and other authors) by plasmids capable of expressing P than does cell killing by P (*trans* P lethality).

P^π^ proteins exhibit lower affinity for DnaB, compared to P [41], which may explain why elevated P^π^ expression at 42 ˚C was needed to evoke the normal P phenotypes in the *grpD55* strain in order for it to compete for and sequester DnaB away from DnaC [11], effectively creating the possibility for a slow-stop type inhibition in *E. coli* replication initiation. A cautionary note here is that the dissociation of DnaB-DnaC complexes by P was only demonstrated *in vitro*.

As well as interacting with λ P [42], DnaB interacts with many cellular proteins, e., g., with DnaA [43], DnaC [44], DnaG [45], SSB [46], the Tau subunit of DNA polymerase III [47], and RNA polymerase [48], and as noted the functional DnaB hexamer is present in limiting amounts, i.e., about 20 hexamers per cell [14,15]. Introducing two missense mutations into *dnaB* will permit the cells to survive constitutive *P* expression (P-lethality), and can suppress P-inhibition of ColE1 replication, yet these mutations do not overtly impede cellular growth/replication. A single missense mutation in *P* can suppress P-lethality and plasmid loss at 37 to 39 ˚C. Therefore, we suggest that an interaction between P and DnaB is an important component of both P-lethality and P-inhibition of ColE1 replication.

Datta *et al*., [28,29] could transform *dnaA rpl* mutants (resistance to *P l*ethality) of the same strain we employed, namely 594, with plasmids expressing P, concluding that an interaction between P and DnaA, and not with DnaB, was important for suppressing P-lethality. Their *dnaA*-rpl8 [29] mutant did not inhibit λ plating [49], and thus has a different phenotype from *E. coli* mutants arising in selections where λ development is inhibited, such as the one used by Saito and Uchida [32]. Sequence analysis revealed that our 594 strain is wild type for *dnaA* [34] and therefore, the *grpD55* mutation in *dnaB* appears sufficient for cellular resistance to P lethality; however, we have not compared the two 594 strains by whole genome sequence analysis. We suggest that these possibly contradictory results are explained by the participation of DnaA in mechanistic events shared with DnaB, allowing the *dnaA*-*rpl* allele(s) to modulate DnaB:P dependent P-lethality and P-inhibition. Alternatively, the reported effect of P protein on DnaA [28] is not at the level of DNA replication, but is in the context of transcription of genes regulated by DnaA.

#### 2.3.1. Model for Plasmid Loss

**Figure 4:**
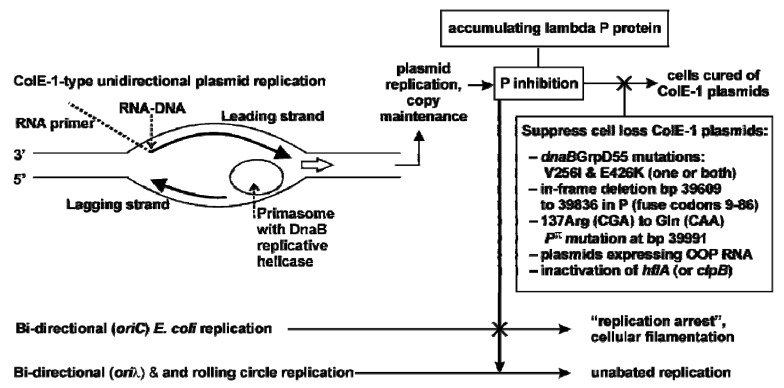
The expression and accumulation of lambda P protein, even in trace levels, interferes with ColE1-type plasmid maintenance replication.

How can P evoke plasmid loss? The expression and accumulation of lambda P protein, even in seemingly trace levels, interferes with ColE1-type plasmid maintenance replication (Figure 4).

Although ColE1 is considered a theta-type replicating plasmid [50], at an early stage, leading-strand synthesis proceeds in the absence of lagging-strand synthesis, yielding unidirectional replication [51,52], and requires DNA polymerase I (Pol I) to initiate continuous leading-strand replication [53,54,55] (shown by open arrow, Figure 4). Replication initiation requires primer RNA II for leading strand synthesis [52,56], stable hybridization of RNA II to DNA [57,58], and processing by RNase H to generate the primer for leading strand synthesis [57,59,60,61]. Pol I extension of the primer unwinds the DNA exposing a primasome assembly signal (n’ pas) or single-strand initiation A sequence (*ssiA*) that allows assembly of the primosome through recruitment and activation of the PriA protein [62]. *This represents a distinct form of DNA replication initiation associated with DNA repair*, in contrast to DnaA-dependent replication initiation at *oriC* [63,64]. Following PriA-primosome assembly, DnaB helicase and DnaG primase are loaded and work coordinately to initiate the discontinuous priming of lagging-strand [58,65], which opens a sequence for termination of lagging-strand synthesis, *terH*, effectively establishing unidirectional replication [53,66]. There is a *dnaA* box that is close to n’ pas, and potentially serves as a DnaA-dependent DnaB-DnaC assembly site [67,68]. The simplest model for ColE1 plasmid replication inhibition and P-dependent plasmid curing is that formation of a cellular P:DnaB complex limits the availability, or perturbs the activity of DnaB to participate in ColE1 replication initiation, perhaps in a step very similar to replication restart of a stalled replication fork, which can involve DnaA. Replication restart proteins form multiple pathways to restart repaired replication forks (Figure 1 in [69]) and they function equivalently to DnaA in forming protein-DNA complexes so that DnaB helicase is loaded onto the DNA [70,71]. Of note, using *in vitro* ΦX174 DNA synthesis assays to monitor DnaB participation, Wickner [2] observed that P inhibited DnaB protein activity in ΦX replication, making a similar proposal to ours for P inhibition of ColE1 replication. 

### 2.4. Influence of P-alteration, or O and OOP RNA Co-Expression on P-lethality and Plasmid Retention

Linking P with a 66 amino acid SPA tag [72] at its C-terminal end did not suppress P lethality, nor plasmid loss, but the lethality of P^SPA^ strongly decreased and plasmid retention sharply increased in the *grpD55* host at 37-42 ˚C (Table 6). The 76 codon in frame N-terminal deletion, *P*^Δ76^, suppressed both plasmid loss and P-lethality at 37 or 39 ˚C. Thus, amino acids 9-86 of P include a critical domain. Its removal knocks out P initiation of replication (our complementation data), the P-lethality phenotype, and P-induced ColE1 plasmid loss.

The allele *P*^π39991^ was used to make pcIpR*-P*^π39991^*-*timm. The mutation was identified in λ*cI*72 mutants that were able to form plaques on the 594 *grpD55* host, and is identical to the πA7 mutation previously sequenced [73]. This R137Q single base change partially suppressed P-lethality and fully suppressed plasmid loss at 37-39 ˚C (but not at 42 ˚C where *P*^π^ was fully expressed) in the *dnaB*^+^ host. The lethality of *P*^π^ significantly increased at 37-39 ˚C in the *grpD55* host even though plasmid retention remained near 100%. Cells with pcIpR*-P*^π39991^*-*timm (or pcIpR*-P­*^SPA^*-*timm) could complement the amber mutation in λ*imm*434*P*am3. 

We asked if co-expression of *O-P*, or if the introduction into the plasmid of the coding sequence for the CII antisense micro-RNA termed OOP, was able to suppress P-lethality and plasmid loss (Table 7).

**Table 7:** Influence of *O*,*P* and OOP-RNA expression on cell killing and plasmid loss. ^a^

Plasmids in 594 host cells	Cell viability and (plasmid retention/cfu; %)at growth temperature (˚C)				
	30	35	37	39	42
pcIpR- *P*-timm	1.0	0.32	0.01	0.008	0.07
	(35/35; 100)	(33/33; 100)	(0/35; 0)	(0/35; 0)	(0/35; 0)
pcIpR- *O-P*-timm	1.0	0.345	0.12	0.12	Nd
	(62/70; 89)	(28/70; 40)	(0/70; 0)	(0/70; 0)	
pcIpR- *O-*36*P*-timm	1.0	0.825	0.793	0.012	Nd
	(30/30; 100)	(30/30; 100)	(30/30; 100)	(1/30; 3)	
pcIpR- *O*-63P-timm	1.0	0.895	0.895	0.055	Nd
	30/30; 100)	(30/30; 100)	(30/30; 100)	(14/40; 35)	
pcIpR- *O*-timm	1.0	1.0	1.0	1.0	0.608
	(30/30; 100)	(30/30; 100)	(30/30;100)	(30/30; 100)	(29/30; 97)
p434’pR- *O*-timm ^b^	1.0	1.0	1.0	1.0	0.615
	(26/30; 87)	(29/30; 97)	(28/30; 93)	(26/30; 87)	(26/30; 87)
pcIpR- *oop*#1-*O-P*-timm ^c^	1.0	0.938	0.20	0.005	Nd
	(120/120; 100)	(120/120; 100)	(98/101; 97)	(115/120; 96)	
pcIpR- *oop*#2 *O-P*-timm^ c^	1.0	0.988	0.055	0.048	Nd
	(117/120; 98)	(60/60; 100)	(76/154; 49)	(62/120; 52)	

^a^: As described for Table 6, except stationary phase cultures were grown up at 30 and not 25 ˚C. ^b^: Plasmid has constitutive expression of *O*. ^c^: The viability results represent the average for four independent plasmid isolates, each single experiments. The results in parentheses, for plasmid retention by survivor cfu, sums results for all the cfu’s assayed from the four isolates. The cell viabilities at 30 and 35 ˚C were at or very near unity. pcIpR-*oop*#1-*O*-*P*-timm: 37˚C (0.34, 0.49, 0.006, 0.008), 39˚C (0.01, 0.006, 0.0006, 0.0015); pcIpR-*oop*#2-*O*-*P*-timm: 37˚C (0.01, 0.01, 0.12, 0.08), and 39˚C (0.01, 0.01, 0.09, 0.08).

The co-expression of *O* would provide an interactive partner for P, possibly lowering the cellular level of unbound P. The 77 nucleotide antisense OOP RNA, which is transcribed from promoter *pO* in the opposite orientation to the *pR-O-P* mRNA (Figure 1A), can hybridize between *pR* and *O*, forming an OOP-RNA: *pR-O-P*-mRNA hybrid that can provide a target for *P* mRNA degradation downstream from the OOP-binding site. The co-expression of *O-P* increased cell viability at 37-39 ˚C, but not plasmid retention. The constitutive or inducible expression of *O* without *P* did not influence cell viability or plasmid loss between 30-39 ˚C. Early studies [74] attempting to determine the coding sequence for *O*, reported a longer protein (O’) made by read-through of the normal stop codon, terminating downstream somewhere within the N-terminal sequence of *P*. Plasmids were made that included the wild type λ sequence between the start of *O* through the UGA stop codon [75], plus 35 or 62 in-frame codons downstream into the *P* DNA sequence, so that if the stop codon for *O* was designated “1”, the next in-frame stop codons would occur at positions 36 or 63 in frame with *O*, even though the downstream reading frame for *P* differs from *O*. While the plasmids expressing the intact *O* sequence were not toxic, the plasmids with O-36P or O-63P, when expressed at 39 ˚C, were toxic and caused plasmid loss. It is unclear if their toxicity depends on O-read-through downstream of its stop codon, or to polypeptides made from the N-terminal end of *P*. Plasmids pcIpR-*oop*#1-*O-P*-timm and pcIpR-*oop*#2-*O-P*-timm (Figure 1A, Table 10), when compared to versions expressing only *P* or *O-P* significantly suppressed plasmid loss at 37-39 ˚C, and partially suppressed P-lethality at 37 ˚C, with the *oop*#1 plasmid showing stronger suppressing effects than the slightly larger #2 version. This data supports a hypothesis that *P* expression from the λ genome is reduced by OOP antisense micro RNA, possibly via a OOP:*pR*-mRNA complex serving as a target for mRNA degradation.

We observed that the co-expression of *O* and *P*, with the potential for formation of an O:P complex, did not suppress the P phenotypes. In contrast, placing the natural *oop-O-P* sequence orientation downstream from *pR* in the pcIpR-[ ]-timm plasmids markedly suppressed P-inhibition of ColE1 replication as monitored by assaying for plasmid curing. This is explained, possibly, by OOP micro-RNA serving in some manner as a competitor to RNA I made by the plasmid, resulting in the loss of plasmid copy control and higher plasmid copies per cell. We view this explanation as unlikely. Alternatively, OOP RNA may limit the adverse effects of *P* expression. It can bind to *pR*-promoted *cII* mRNA and target its degradation in an RNaseIII-dependent reaction [76,77], suggested to limit downstream *O-P* gene expression.

What is the possibility that inefficient termination of *pR*-GOI transcription and consequent run-on transcription could cause problems with plasmid maintenance and explain the loss of pcIpR-*P*-timm from cells, rather than a direct effect of P on plasmid maintenance? We have observed that the wild type *timm* terminator prevents read through from the *cI-rex* transcript into the *oL* operator region or through into *N* when CII stimulated transcription from *pE* occurs at 30 to 100-fold the level of *cI-rex* transcription from the *cI* maintence promoter *pM* (see [17] and contained references). This was the rationale for incorporating *timm* into the design of pcIpR-(GOI)-timm plasmids. Table 7 shows the influence of expression of lambda gene *O* from plasmids pcIpR-*O*-timm and p434’pR-*O*-timm on plasmid loss at 30, 35, 37, 39 and 42 ˚C. The immunity properties of these two plasmids was shown in Table 4 of [36]. The expression of gene O is constitutive at all temperatures from plasmid p434’pR-*O*-timm. It can be seen that 100% cellular maintenance / retention occurred for plasmid pcIpR-*O*-timm between 30-39 ˚C and 97% at 42 ˚C. Even with constitutive expression of *O* from p434’pR-*O*-timm over the range of cell growth temperatures, between 87-97% of the cells retained the plasmid. A similar result is also seen in Table 6 for plasmid pcIpR-*P*^Δ76^-timm, where 99% of the cells grown between 25 and 42 ˚C retained the plasmid. These results strongly support an argument that *timm* in pcIpR-(GOI)-timm is likely a powerful terminator that prevents inefficient termination and consequent run-on transcription, which might otherwise cause problems with plasmid maintenance.

### 2.5. Replicative (*cis*) killing; P (*trans*) Lethality / Inhibition

We compared the kinetics of cell death resulting from *P* expression in cells with pcIpR*-P-*timm, i.e., P-lethality (Table 8A) with Replicative Killing, resulting from initiating *ori*λ replication from the Y836 chromosome (Figure 1C) or in cells where this fragment was moved by transduction (Table 8B). Shifting these cell cultures to 42 ˚C denatures the reversibly-denaturable CI[Ts]857 repressor [17,78] resulting in transcription from promoter *pR*, and *P* expression from the plasmid, or *cro-cII-O-P-ren* expression from the chromosomally inserted λ fragment. The latter event results in the initiation of bidirectional replication from *ori*λ. After the period of gene de-repression the cells were swirled in an ice bath and then plated for survivors at 30 ˚C, which serves to shut off transcription from *pR* via the renaturation of CI, which, in turn stimulates *cI* expression.

The λ fragment is not excised from the chromosome of strain Y836 or from the (*cIII-ren*)λ-transduced 594 variant since each inserted λ fragment lacks the genes for *int-xis-kil* and is deleted for both *attL* and *attR* sites. The *ori*λ initiation event from these integrated λ fragments exerts a rapid *cis*-active Replicative Killing effect, likely causing interference with *E. coli* replication fork progression. In *cis*-killing 75-87% of the cells die (i.e., are not rescued upon spreading / incubating the cells at 30 ˚C) by 20 min after prophage induction (Table 8B). But the *trans*-P lethality/inhibition effect is reversible, to a point, and much slower as only 14% of the cells are killed after 1 hr (55% after 2 hr) of *P* expression, even though >90% of the cells retained the plasmid (Table 8A).

**Table 8:** Contrasting *trans* P-lethality / inhibition and *cis* Replicative Killing

**A. ** **Cells tolerate short-term exposure to P (short-term interference, not lethality) ^a^**			
Strain with plasmid	Incubation at 37 ˚C	Cell viability (Amp^R^ cfu/ total cfu)	Outcome of P expression from plasmid
594[pcIpR-*P*-timm]	1 hr	0.87 (232/242)	most cells recover
	2 hr	0.49 (258/281)	many cells recover
	6 hr	0.13 (2/173)	high plasmid loss
**B. ** **Inducing replication from a trapped cryptic prophage causes Replicative Killing^ a^**			
Strains with cryptic prophages	Prophage Induction time	Cell viability	Outcome of prophage Induction
Y836[~*cIII-cI*857*-O-P-ren*]	5 min	0.33	rapid cell killing
	20 min	0.13	rapid cell killing
	3 hr	0.00018	extensive cell killing
	5 hr	0.00008	extensive cell killing
594[~*cIII-cI*857*-O-P-ren*]	5 min	0.55	rapid cell killing
	20 min	0.25	rapid cell killing
	3 hr	0.0022	extensive cell killing
	5 hr	0.0022	extensive cell killing
Y836[~*cIII-cI*857*-O-P::kan-ren*]	3 hr	5.1 ^b^	cell growth
	5 hr	6.1 ^b^	cell growth

^a^: Refer to Experimental Section *3.6*. ^b^: The increase in viability by for example 5.1 indicates somewhat more than two and less than three cell doublings.

#### 2.5.1. Contrasting *trans* P-lethality / inhibition, and *cis* Replicative Killing

We showed that shifting culture cells transformed with pcIpR-*P*-timm and grown at 25 ˚C up to 37 ˚C for two hrs reduced cell viability to about half, and thus cells are capable of tolerating or metabolizing some level of P, with a reported half-life of up to an hr [79,80]. However, initiating irreversible *ori*λ replication from a trapped cryptic prophage, Figure 1C, reduced cell viability between 4 and 10-fold after only 20 min of induction. The Replicative Killing (RK^+^ phenotype) survivor frequency of 10^-6^ to 10^-8^ is distinguished by rare RK^-^ mutants in host or prophage (not merely in *P*) that block some aspect of λ replication initiation [78,81,82,83]. We suggest the terms *cis* Replicative Killing and *trans* P lethality to distinguish mechanistically these the two ideas. The RK^+^ phenotype seems uniquely dependent upon multiple, non-repairable λ replication forks arising from *ori*λ (drawn in Figure 1C), but there are likely multiple possibilities for P-lethality. Several experiments suggest that the encoded gene *ren* downstream of *P* is not responsible for P-lethality. A modified version of pHB30 (i.e., pHB31) encoding *P-ren,* but deleted for bases 39609-39836 within *P* (equivalent to *P*^Δ76^ used herein) was fully capable of transforming 594 cells at 42 ˚C, suggesting that P, not Ren blocked transformation at 42 ˚C [31]. RK^-^ mutants of Y836 with insertions or deletions (recombinant or natural) within/inactivating *O* (ilr208b, ilr223a, ilr541c, ilr200b, ilr203b, ilr207d, ilr201b), or *P* (ilr566a, Bib11t), yet which sequenced to be *ren*^+^, lost the Replicative Killing competence phenotype when shifted from 30 to 42 ˚C, suggesting that replication initiation from *ori*λ, and not *ren* expression, is responsible for Replicative Killing; transduction of the λ fragments into 594 did not alter the phenotype observed for Y836 [RK^+^], or the RK^-^ mutants. Ren is not required for P-lethality phenotype, nor for Replicative Killing, i.e., in the absence of *ori*λ replication initiation, *ren* expression will not produce the RK^+^ phenotype, however, our experiments do not rule out some ancillary role for Ren.

Several examples reported suggest that P-lethality is a separate mechanism from P-inhibition of ColE1 replication: i) *grpD55* cells with pcIpR-*P*^π^-timm were killed at 37–39 ˚C, yet 100% of the surviving cells retained the plasmid; ii) most of the cells with pcIpR-*oop-O-P*-timm plasmids retained the plasmids at 39 ˚C while suffering high P-lethality. Additionally (see Figure 4), cells defective in host protease genes *clpB* or *hflA* were sensitive to P lethality at 37-39 ˚C, yet the majority of the survivors retained the pcIpR-*P*-timm plasmid (data not shown; available from authors).

Is P-inhibition due only to a decrease in available DnaB, or does it influence / perturb ongoing replication, or replication restart? Our results do not indicate if P fully sequesters DnaB or if a single P monomer bound to DnaB is sufficient. Moreover, the genetic evidence does not show whether this interaction is persistent or transient. There seems little doubt that competing DnaB away from DnaC is an important function of P; but the longer term consequence is that the interaction of P and DnaB yields a P-DnaB-ATP dead-end ternary complex [10], and the only known role for P is in early *ori*λ-dependent initiation events, of which only a few are required. We propose that the full sequestration of DnaB by P is not necessary in order for P to interfere with ongoing plasmid replication, because of the sensitivity of ColE1 replication / copy maintenance to leaky (sub-induction) levels of P (arising from a plasmid encoding *P*). The simple explanation for plasmid loss is that while the cells continue to replicate and divide, plasmid replication is differentially inhibited. For example, *grpD55* cells very poorly support vegetative growth of λ at 30 ˚C, and not at all between 37–42 ˚C, whereas cellular growth is not noticeably perturbed. A low level of P, tolerated by cells, could fully inhibit ColE1 replication, in turn reducing plasmid copy number with each cell division, which could explain plasmid curing for cells grown between 34–36 ˚C. Less clear is how cells with pcIpR-*P*-timm grown at 30 ˚C are cured when shifted to 37–42 ˚C, where division is inhibited and cells elongate forming filaments (Sect 2.8.).

### 2.6. Does P Expression from pcIpR-P-timm perturb λ Vegetative Growth?

As previously noted, although present in very limited amounts in the cell, DnaB is a multifunctional protein involved in replication fork movement [3], serving as a mobile promoter in priming reactions [4,5], in the progression of Holliday junctions [6,84], and in replication restart reactions [70,71], some of which are likely involved in λ replication beyond the *ori*λ-O-P-dependent initiation step. Since the interaction of P and DnaB yields a P-DnaB-ATP dead-end ternary complex [10], we wondered if the expression of P from fully induced pcIpR-*P*-timm in cells shifted to 42 ˚C could serve to limit λ replication, as monitored by phage burst. Lysogenic cultures with a λ*cI*[Ts]857 *S*am7 prophage defective for natural cell lysis (because of the nonsense mutation in *S*) were synchronously induced by shifting the cells from 30 to 42 ˚C. One of the parallel cultures contained pcIpR-*P*-timm. Following induction, the cells were artificially lysed. Relative phage burst from each of the cultures was determined by dividing the released phage titer by the cell titer pre-induction. The cells with pcIpR-*P*-timm showed an increased relative burst by 60 min post induction over parallel cells without the plasmid (i.e., bursts of 248 (cells with pcIpR-*P*-timm) and 42). Clearly, λ replication / maturation is not curtailed (and appears enhanced) by combined expression of *P* from both induced prophage and plasmid, which is dramatically opposite to the effect of *P* expression on ColE1 plasmid replication/maintenance.

#### 2.6.1. λ Replication and Phage Maturation

Our results suggest that P is not inhibitory to λ replication, which, considering its influence on *E. coli* and ColE1 replication, raises some interesting questions. Doesn’t λ need to deal with replication restart, likely one of several possible targets for P-DnaB interaction, or does it have an alternative mechanism? Does P or a P-complex have an unrecognized DNA helicase activity? While some *P* analogues of lambdoid-type phages encode their own DNA helicase activity (see suppl. Figure S2 in [36], and [85] for comparison of 20 *P*-like genes in lambdoid phages), P is not recognized as having this property and those P-like proteins with putative helicase activities are larger than λ *P*.

### 2.7. P-Induced Cellular Filamentation

**Figure 5:**
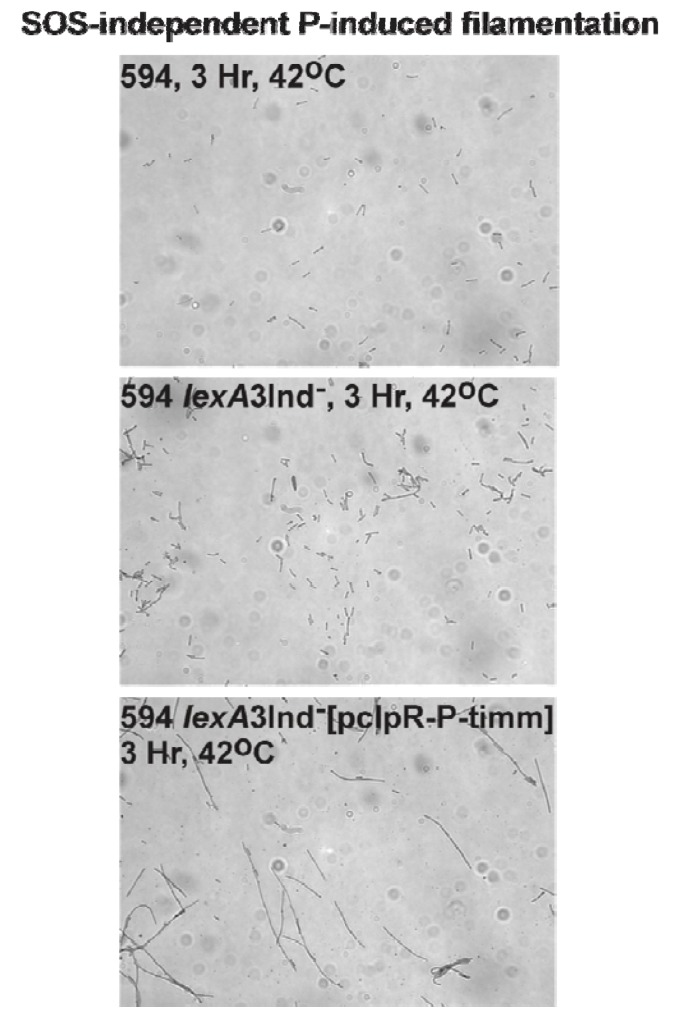
Cellular filamentation resulting from induced P-expression. The photos are representative of data shown for one of the three sets of photos per strain per assay condition that were used for cell measurements in Table 9.

We induced *P* expression from plasmids pHB30 and pcIpR*-P-*timm and followed filament formation (Table 9) in SOS^+^ strain 594, and in SOS-defective variants [86] of 594 made by transducing in the alleles of *lexA*3[Ind^-^], where the alanine-glycine protease cleavage site [87] is changed to alanine-aspartate [88] (Figure 5, Table 9), or Δ*recA* (Table 9)

The results in Table 9 suggest that *P* expression negatively impacts *E. coli* cell division. The cellular filamentation observed upon shifting 594 *lexA*3[pcIpR-*P*-timm] cells to 37 ˚C (Table 9) supports the concept (e.g., from Figure 3) that some leaky *P* expression arises from this plasmid at 37 ˚C in sufficient level to prevent ColE1 plasmid replication maintenance.

**Table 9:** SOS-independent P-induced cellular filamentation.^ a^

Strain [plasmid]	Time(Temp)	Relative cell length										Sumcells
		1X	2X	3X	4X	5X	6X	7X	8X	9X	≥10X	
594	0 (25)	26	19	0	0	0	0	0	0	0	0	45
“	1 (42)	15	11	11	8	0	0	0	0	0	0	45
“	3 (42)	28	15	2	0	0	0	0	0	0	0	45
“	5 (42)	29	14	2	0	0	0	0	0	0	0	45
594 *lexA*3	0 (25)	40	32	2	0	0	0	0	0	0	0	74
“	1 (42)	23	16	6	0	0	0	0	0	0	0	45
“	3 (42)	31	13	1	0	0	0	0	0	0	0	45
“	5 (42)	42	3	0	0	0	0	0	0	0	0	45
594 *lexA3*[pcIpR-*P*-timm]	0 (25)	71	19	0	0	0	0	0	0	0	0	90
“	1 (37)	21	23	1	0	0	0	0	0	0	0	45
“	3 (37)	7	9	9	6	4	3	2	1	2	2	45
“	5 (37)	6	20	0	0	2	0	3	3	3	8	45
594 *lexA3*[pcIpR-*P*-timm]	1 (42)	9	19	13	3	1	0	0	0	0	0	45
“	3 (42)	11	1	3	4	4	9	8	2	2	1	45
“	5 (42)	8	6	7	1	1	0	2	1	2	14	42
594 [pHB30]	0 (30)	6	20	4	0	0	0	0	0	0	0	30
“	1 (42)	0	2	10	9	3	4	1	1	0	0	30
“	3 (42)	2	4	8	3	2	5	2	1	2	1	30
“	5 (42)	2	8	10	2	2	2	3	0	0	1	30
594 *lexA* [pHB30]	0 (30)	5	17	8	0	0	0	0	0	0	0	30
“	1 (42)	0	6	6	8	4	1	4	0	1	0	30
“	3 (42)	3	5	2	6	4	0	5	3	1	1	30
“	5 (42)	1	7	5	6	5	2	1	1	0	2	30
594 Δ*recA*	0 (30)	16	12	2	0	0	0	0	0	0	0	30
“	1 (42)	4	12	7	4	1	2	0	0	0	0	30
“	3 (42)	9	16	2	4	2	0	0	0	0	0	30
“	5 (42)	12	11	4	2	0	0	1	0	0	0	30
594 Δ*recA* [pHB30]	0 (30)	8	17	4	1	0	0	0	0	0	0	30
“	1 (42)	2	5	8	10	3	1	1	0	0	0	30
“	3 (42)	5	4	5	3	6	4	2	0	0	1	30
“	5 (42)	3	5	4	8	0	4	2	0	1	3	30

^a^: Refer to Experimental Section *3.7*.

#### 2.7.1. P-Dependent Cellular Filamentation

We previously observed extensive cellular filamentation upon the de-repression of λ*cI*[Ts]857*O*am8 prophage, but not for a λ*cI*[Ts]857*P*am3 prophage in lysogenic cells [31]. Klinkert and Klein [23] demonstrated that expression of *P* from a plasmid can cause cellular filamentation, which they attributed to an ability of P to impair bacterial DNA synthesis. Agents that block cellular DNA synthesis can stimulate a cellular SOS response [86], which in turn elevates expression of one of the SOS response genes, *sulA*, whose product prevents the action of FtsZ at the site of septum formation, causing a cell to stop dividing [89,90], and allows time for DNA repair (see [91]). Cells inhibited for FtsZ activity form filaments that are longer than dividing cells. Induction defective mutations in *lexA* encoding the repressor for the SOS response, or certain *recA* mutations prevent the induction of a cellular SOS response [86].

The hypothesis that P-lethality does not involve an interaction between P and DnaB, but is instead targeted to DnaA, implies that the P:DnaA interaction can prevent *E. coli* replication initiation at *oriC* by removing available DnaA. Previous studies have shown that a *dnaA*46[Ts] mutant exhibits a slow-stop effect on DNA synthesis when the cells are shifted to 42 ˚C, blocking re-initiation at *oriC*; however, these cells continue to divide, forming anucleate cells, not filaments [92,93]. Other cellular interactions beyond those between DnaA or DnaB and P are important. For example, as noted, P interacts with DnaK and DnaJ. The inactivation of *dnaK*, and apparently *dnaJ*, results in multiple cellular defects, including formation of cell filaments, abnormally segregated chromosomes, and loss of plasmid maintenance [94].

Does P function in replication restart? It was reported [69] that neither *lexA* nor *sulA* deletions abolished filamentation in a *priA sulB* mutant, suggesting models whereby repair/restart of chromosomal replication is essential for completing a round of replication, and that replicative forms are resolved to monomers before cell division can take place. Since DnaB is reloaded onto DNA for replication restart, P accumulation could serve to limit the availability of DnaB to replication restart pathways, or it may directly interact with the elongating replisome, neither previously suggested. The need for replication restart is influenced by collisions between transcription and replication forks. RNA polymerase mutations in *rpoB* and *rpoC* [95,96] facilitate replication progression. Indeed, CH has shown that two of 22 mutants obtained in screens for rifampicin-resistant *E. coli* cfu had significant resistance to P-lethality (remainder did not; Hayes lab, unpublished results). However, among the group of Rif^R^ isolates, 30 were localized by DNA sequence analysis to two regions within *rpoB* (because *rpoB* is 4029 bp, we did not sequence the whole gene), but the site of mutation in the two resistant isolates remains undetermined, and the existence of additional suppressor mutations arising in these two mutants remains a possibility. We predict that mutations in *E. coli* which decrease conflicts between transcription and replication would make cells more resistant to P, and mutations which increase conflicts would make cells more sensitive.

## 3. Experimental Section

### 3.1. Strains Employed

The bacteria, plasmids (Figure 1A) and phages employed are listed in Table 10.

**Table 10:** Bacteria, plasmids and phages employed.

Bacterial strains	Characteristics or genotype	Source/Ref.’; Hayes lab #^a^
594	F^-^*lac*-3350 *galK*2 *galT*22 *rpsL*179 IN(*rrnD*-*rrnE*)1; see [97]; called R594	[97], SH lab; B10
TC600	*supE*, Pm^+^	SH lab, B8
Ymel	*supF*, Pm^+^	SH lab, B71
DE407	*lexA*3[Ind^-^] *malB*::Tn*9* Tet^R^*sulA*211 *sfiA*11, UV^S^	D. Ennis; B142
FC40 (=SMR624)	Δ(*srlR*-*recA*)306::Tn*10* Tet^R^ UV^S^	SM Rosenberg [98]; Y921
AB2834 *aroE*	*grpD55*, *thi* tsx^R^ λ^R^ at 42 ˚C from K552	H. Uchida [32,33]; NB83
W3874 *malB*5	*dnaB grpA80* *lac*^-^ Str^R^ λ^R^ at 42 ˚C	[32], NB81
W3350 *dnaB*-*grpD55*	*grpD55* *malF*3089::Tn*10* Tet^R^λ^R^ at 42°C, λ*rep*P22^S^	[33], NB15
594 *dnaB*-*grpD55*	*grpD55* allele *malF*3089::Tn*10*; Tet^R^, λ^R^ at 42°C, λ*rep*P22^S^	[34], NB295
594 *lexA*3[Ind^-^] *malB*::Tn*9*	LexA repressor induction defective	CE, NB293
594 Δ(*srlR-recA*)306::Tn*10*	deletion of *recA* Tet^R^ UV^S^	CE, B318
W3350	*F^-^ lac^-^*3350 *galK*2 *galT*22 *rpsL*179 IN(*rrnD*-*rrnE*)1	SH lab, B12
Y836	SA500(λ*bio*275*cI*[Ts]857 Δ431) *his^-^*	[78,82], NY1049
594::*nadA*::Tn*10* [~*cIII*-*ren*]^λ^	Tn*10* [zbh29 at 16.8 min] *bio^+^* transductant = 594 *bio275* (λ*cIII*-*cI*[Ts]857-O-P-*ren*) Δ431	A. Chu, SH lab, NY1057
Y836 *P*::kan(Bib11t)	SA500 (λ*bio*275 *cI*[Ts]857 *O^+^P*::kan Δ431) *his^- ^*Kan^R^	SH, NY1153
594(λ*cI*857*S*am7)	λ lysogen defective for cell lysis	C. Marek, SH lab, Y1163
594(λ*cI*857*S*am7)[pcIpR*-P-*timm]	as above with transformed plasmid	SH lab, P509
594 *clpP*::kan	*clpP*^-^, Kan^R^ from SG22159	S. Gottesman; [99], NB276
**Plasmids **	**Transformed into strain 594**	**Source/Ref.’; Hayes lab #^a^**
pUC19	Wild type Amp^R^ (New England Biolabs)	NP188
pcIpR-*P*-timm	*Bam*HI-*Cla*I PCR fragment from λcI857, replacing D-CAP in P459 with λ bp’s 39582-40280	CH, P466
pcIpR-*P*::kan-timm	PCR *Bam*HI-*Cla*I fragment from Y836 *P*::kan(Bib11t) strain NY1153	KM, P510
PcIpR-*P-*SPA*-*timm ^b^	Replace D in P462 between *Bam*HI and AscI sites with *Bam*HI-P(λ bp’s 39582-40280)-*Asc*I PCR fragment	KM, P467
pcIpR-*P*^Δ76^-timm	In-frame deletion76 codons: λbp 39609-39836 in pcIpR-*P*-timm with HpaI, ligate	KM, P515
pcIpR-*P*^π^-timm	*Bam*HI-*Cla*I PCR fragment from λcI72π Lysate #3a, replacing D-CAP in P459 with λ bp’s 39582-40280	KM, P505
pcIpR-*O*-timm	*Bam*HI-*Cla*I PCR fragment from λcI857, replacing D-CAP in P459 with λ bp’s 38686-39582	[36], CH, P465
P434’pR-*O*-timm	Constitutive *O* expression; *Bam*HI-*Cla*I PCR fragment from λcI857, replacing D-CAP in P459 with λ bp’s 38686-39582	[36], CH, P494
pcIpR-*O-P*-timm	*Bam*HI-*Cla*I PCR fragment from λcI857, replacing D-CAP in P459 with λ bp’s 38686-40280	CH, P569
pcIpR-*O-*36*P*-timm	*Bam*HI-*Cla*I PCR fragment from λcI857, replacing D-CAP in P459 with λ bp’s 38686-39687	CH, P565
pcIpR-*O-*63*P*-timm	*Bam*HI-*Cla*I PCR fragment from λcI857, replacing D-CAP in P459 with λ bp’s 38686-39768	CH, P566
pcIpR-*oop*#1-*O-P*-timm	*Bam*HI-*Cla*I PCR fragment from λcI857, replacing D-CAP in P459 with λ bp’s 38559-40280	CH, P567
pcIpR-*oop*#2-*O-P*-timm	*Bam*HI-*Cla*I PCR fragment from λcI857, replacing D-CAP in P459 with λ bp’s 38546-40280	CH, P568
pHB30	λ bases 34499-34696, 36965-38103, 38814-40806 (see Section 3.2.)	[31,34], SH lab, P8
**Bacteriophage **	**Genotype**	**Hayes lab lysate #**
λ wild type (wt)	λpapa	[78], 944,1001
λ*cI*72	*cI^-^*	[78], 951, 999
λnin5	made from λ wt	[78], CH, 698
Λvir	λ*v2v1v3*	[78], 260
λ*cI*857	*cI*[Ts]857	[100], 1002
λ*cI*857*S*am7	defective for cell lysis	[101], 963
λ*imm*434*P*am3	*imm*434, sequenced *P*am3 mutation C to T, λ base 39786 (CAG to TAG)	[83], SH lab, 518, 664
λ*imm*434nin5	*imm*434, Δnin5 region, forms very turbid plaques at 37˚C	[22], CH, 963

^a^: The strain numbers are from the Hayes laboratory collections. All gene inserts within the pcIpR-[ ]-timm plasmids were sequenced to confirm the genetic integrity of the inserted fragment. ^b^: Plasmid pcIpR-D-SPA-timm [20] (strain P462) was prepared from pcIpR-D-CAP-timm (strain P459), replacing 318 bp CAP from P459 by digestion with *Asc*I and *Cla*I and replacing with 239 bp SPA tag from pMZS3F [72] (from J. Greenblatt) isolated via PCR with primers L-Asc-CBP & R-ClaI-FLAG. SPA is a 66 amino acid tag with 3X FLAG sequences.

### 3.2. Construction of Expression Vectors for λ Genes, Gel Analysis of Plasmids, Insertion Localization

The plasmids were extracted from cells using Qiagen plasmid mini preps. They were separated by agarose electrophoresis on gels made and run using 1X TBE buffer (10X = 1M Tris, 1M Boric acid, 0.02 M Na_2_EDTA, pH 8). The precursor plasmid pcIpR was made by degrading pBR322 with *Eco*RI and *Bam*HI and purifying the large *Eco*RI-*bla*-rep/rop-*Bam*HI fragment. This was ligated with an 833 bp PCR fragment derived from λ*cI*857 DNA amplified using primers that added MfeI and *Bam*HI tags to the ends of lambda bases 37203 and 38036 to produce plasmid pcIpR, where the ligation of the MfeI end to the *Eco*RI site removed both sites in the resulting construct. pcIpR was digested with *Bam*HI and SalI and the large fragment of 4817 bp was separated from the 276 bp region between the *Bam*HI and SalI sites. A synthetic DNA sequence including λ oR/pR region, a *Bam*HI site, gene fusion D-CAP-*Cla*I-timm-*Eco*RI-SalI sequence provided by IDT, Coralville IA, was digested with *Bam*HI and SalI and the 710 bp fragment was ligated to the 4817 bp fragment to produce pcIpR-D-CAP-timm [20]. This permitted positioning the AUG for the D-CAP fusion protein immediately to the right of the *Bam*HI sequence, and blended into the consensus Shine-Delgarno sequence, so that the D-fusion orf was positioned 18 bp downstream from the mRNA start site for the λ *pR* promoter (see Figure 1, [20]). The D-CAP orf was removed from pcIpR-D-CAP-timm λ by digestion with *Bam*HI and *Cla*I and the resulting large fragment was ligated with gene *P*, produced by generating a *Bam*HI-*P*-*Cla*I PCR fragment, including the precise sequence of *P* (λ bp’s 39582 – 40280) from λ*cI*857 DNA to yield pcIpR-*P*-timm, where a TAA stop codon was added that immediately followed the *P* insertion. Plasmid pcIpR-*P*^π-39991^-timm was generated by inserting a *Bam*HI-*P*^π^-*Cla*I PCR fragment made from λ*cI*72-*P*^π-39991^ phage. The plasmid pcIpR-*P*^Δ76^-timm was constructed by using restriction endonuclease *Hpa*I to delete λ bp 39609–39836, i.e., bp 28 through 255 within the N-terminal end of *P,* then ligating to fuse codon 9 with codon 86. The plasmid pcIpR-*P*-SPA-timm was constructed by removing the *Bam*HI to AscI fragment from the 5155 bp plasmid pcIpR-D-SPA-timm [20], and ligating with the remaining 4807 bp fragment a *Bam*HI-*P*-AscI PCR fragment encoding lambda bases 39582 to 40280 to produce the 5524 bp plasmid pcIpR-*P*-SPA-timm. SPA is a 66 amino acid tag sequence [72] with both calmodulin and 3XFLAG binding recognition sequences. Plasmids pcIpR-*O*-timm and p434’pR-*O*-timm have the precise *O* sequence (ATG=38686-39582) plus TAA stop codon. In p434’pR-*O*-timm, the SD differed by one bp compared to SD in pcIpR-*O*-timm because of the slightly different sequence ahead of *cro* in *imm*434 DNA [102]. Plasmids pcIpR-*O*-36P-timm and pcIpR-*O*-63P-timm, respectively, have λ DNA sequences 38686–39687, or 38686–39768, each including an intact *O* sequence plus an extension comprising the N-terminal portion of *P*, followed by TAA stop at the end of inserted partial *P* sequence. Plasmids pcIpR-*O-P*-timm (with precise *O-P* sequence), pcIpR-*oop*#1*-O-P-*timm and pcIpR-*oop*#2*-O-P-*timm (each with DNA from within *cII* through *P*) have inserted sequences, respectively: 38686-40280, 38559-40280, and 38546-40280, followed by TAA stop codon. All GOI inserts within the pcIpR-[GOI]-timm plasmids were sequenced to confirm the genetic integrity of the inserted fragment. pHB30 [31] contains λ genes *cI*[Ts]857, a *cro-O* in frame fusion, *P-ren*; i.e., the pBR322 bases from 375–4286 and λ bases (*Bam*HI)34499-34696(*Cla*I)-(*Cla*I)36965-38103(BglII)-(BglII)38814-40806(AatII) and was re-characterized and sequenced [34].

### 3.3. Plasmid Transformation; Phage and Culture Assays

Cells from a single colony of the *E. coli* strain being transformed with a plasmid were inoculated into 20 ml fresh LB (5 g NaCl, 10g Bacto Tryptone, 10 g Bacto Yeast Extract per liter), grown overnight, subcultured into fresh LB medium and grown at 30 ˚C to A_575_ = 0.4, which equals about 4 × 10^8^ cfu (colony forming units) per ml. The cells (1 ml) were centrifuged in 1.5ml microtubes for 1 min at 12 × 10^3^ rpm in an Eppendorf 5424 microcentrifuge. The supernatant was decanted and cell pellet washed with 750ul 0.01M NaCl. The cells were again pelleted, suspended in 750 µl ice cold 0.03 M CaCl_2_, incubated on ice for 30 min, pelleted, and resuspended in 150 ul 0.03 M CaCl_2_ = competent cells. 200 ng DNA of the plasmid in TE* buffer (0.01 M Tris, 0.001 M Na_2_EDTA, pH 7.6) was combined with the competent cells and mixed gently. The mixture was held on ice for 60 min, the tubes were heat shocked at 42 ˚C for 90 seconds in a heating block and plunged on ice for 2 min. 850 ul of room temperature LB was added to each sample tube and these were incubated with gentle shaking in a 25 ˚C water bath for 90 min. At the end of incubation time the cell samples were diluted in ø80 buffer (1.2 g Tris, 5.8 g NaCl per liter, pH 7.6). Then aliquots (0.1 ml) were spread on LB or TB (10 g Bacto tryptone, 5 g NaCl per liter) agar plates. The TB or LB medium used for the solid support agar plates included the addition of 11 g Bacto agar per liter prior to autoclaving. The agar plates used for screening Amp^R^ cfu were supplemented with 50 μg/ml of ampicillin (=Amp50) added after the autoclaved agar medium had cooled, prior to pouring the plates. Molten TB top agar (10 g Bacto tryptone, 5 g NaCl and 6.5 g Bacto agar per liter) was used for plating phage.

### 3.4. Sequence Analysis of Alleles of dnaB

The *dnaB* genes were amplified with primers DnaB-1 and DnaB-6. The PCR fragments were sequenced with overlapping primer pairs DnaB-1 and DnaB-2, DnaB-3 and DnaB-4, and DnaB-5 and DnaB-6 [34]. The *grpA80* and *grpD55* GenBank accession nos. are DQ324464 and DQ324465.

### 3.5. Assessing Influence of Modifying P or dnaB on P Lethality and Plasmid Loss

The cultures were grown up to stationary phase in TB culture medium plus 50µg/ml ampicillin for 48 hr at 25 ˚C, diluted, spread on TB agar plates that were incubated at 25, 30, 37, 39, or 42 ˚C for 48 hr and cfu per ml was determined. Survivor cfu arising on the plates were stabbed to TB and TB+Amp50 plates to estimate the proportion of cfu retaining the plasmid. We tried to assay all cfu per dilution plate sector(s) to avoid colony size discrimination. Individual cfu arising from 594 *grpD55* [pcIpR-*P*-timm] from the 25 or 42 ˚C plates (single colonies – sc1 through sc6) were inoculated into TB+Amp50 plates, incubated 48 hrs at 25 ˚C, and plasmid DNA was extracted. The extracted plasmid preparations from the six individual cultures were each transformed into 594 culture cells and Amp^R^ cfu were selected on agar plates incubated at 25 ˚C. Individual single colonies (sc’s 1-6) from these plates were inoculated into TB with 50 µg/ml ampicillin and grown 48 hr at 25 ˚C, then diluted and spread on TB agar plates that were incubated at 25, 37, 39, or 42 ˚C for 48 hr and cfu per ml was determined. Survivor cfu arising on the plates were stabbed to TB and TB+Amp50 plates to estimate the proportion of cfu retaining the plasmid. The experiment with the *re-claimed* plasmids was undertaken to determine if the survivor plasmids extracted from the 594 *grpD55* [pcIpR-*P*-timm] cultures grown at 42 ˚C retained the P-lethality phenotype.

### 3.6. Contrasting *trans* P-Lethality / Inhibition and *cis* Replicative Killing

The influence of transient *P* expression (*trans* P lethality) from pcIpR-*P*-timm on cell viability and plasmid loss was assessed by incubating cells diluted and spread on pre-heated agar plates, that were held at 37 ˚C for 1, 2, or 6 hr, and then incubated at 25 ˚C for about 72 hrs for survivor cfu. The survivors were stabbed to TB+Amp50 agar plates to assess for plasmid loss. Replicative Killing, or *cis* killing, involves the irreversible effect of inducing gene expression from a trapped (nonexcisable) cryptic fragment of λ [~*cIII-ren* genes in Y836] inserted within bacterial chromosome. The result is the formation of an onion-skin replication bubble at *ori*λ site in bacterial chromosome as shown in Figure 1C. The λ fragment in strain Y836 was transduced into 594 in two steps by P1 transduction: a) moving *nadA*::Tn*10* into Y836, and b) moving the *nadA*::Tn*10* [~*cIII-cI*857*-O-P-ren*]^λ^ fragment into 594. Gene *P* expression in strain Y836 was inactivated by recombineering involving use of primers L-P-stop-kan [5’-gaccgtgagcagatgcgtcggatcgccaacaacatgactaactagctctgatgttacattgcacaag] and R-kan-stop-P [5’-ggtcgattctgccgacgggctacgcgcattcctgcgctagttagtcagtcagcgtaatgctctgcca] and the insertion of Kan^R^ (same as in pBR322::kan) into *P* to make strain Y836 [~*cIII-cI*857*-O-P*::kan*-ren*] (isolate Bib11t), where Kan substituted the bases 39651-39838 of *P*.

### 3.7. SOS-Independent P-Induced Cellular Filamentation

Overnight cultures of each strain were prepared in TB (plus 50 μg/ml ampicillin for strains with plasmids) and grown at 30 ˚C for the controls and strains with pHB30, and at 25 ˚C for strains with pcIpR-P-timm. Subcultures were made into 50 ml TB or TB-Amp50 and grown at 25 or 30 ˚C, as shown for 0-time, to an A575nm of 0.01 to 0.15. An aliquot was removed for the 0-time assay and the flasks were transferred to 37 or 42 ˚C shaking water baths. For each assay point culture aliquots were removed (5 μl, and 1 μl then diluted with 4 μl φ80 buffer) and placed on glass slides. The samples were allowed to air dry and gently fixed over a flame. The slides were prepared by Gram stain, and then examined by light microscopy, taking three pictures per slide of areas with lower density cells. The digital pictures were projected onto a large screen and the length per cell was manually measured, 15 cells per slide. Representative culture absorbance 0 time, 1, 3 and 5 hrs at inducing temperature: 594 (0.09, 0.22, 0.74, 0.95); 594 lexA3 (0.01, 0.25, 0.68, 0.95); 594 lexA3[pcIpR-P-timm] induced to 37 ˚C (0.10, 0.26, 0.54, 0.76); and 594 lexA3[pcIpR-P-timm] induced to 42 ˚C (0.10, 0.25, 0.50, 0.67). The value for 1X represented the average of ten smallest cell measurements for 594 cells, representing 35.6 mm. All values were rounded up into next category, e.g., cells with length >53.4 mm were presented as 2X average length.

### 3.8. P-Induced Plasmid Loss

The volume of cells utilized for plasmid extraction from each of the cultures was normalized to achieve a final A_575_ of 1.0. Aliquots were removed to extract plasmid DNA and isolate cfu on TB agar plates. The survivor cfu arising on the TB plates were stabbed to TB and TB+Amp50 plates to estimate the proportion of cfu retaining the Amp^R^ plasmid. Plasmid DNA was extracted from aliquots (5 ml) of the cultures and mixed with 0.5 ml of a stationary phase culture of 594[pUC19] (serving as an internal plasmid extraction / gel loading control). DNA was extracted from the cell pellet(s) using QIAgen spin mimiprep kits and suspended in 0.05 ml elution buffer. Aliquots from all DNA preparations were digested to completion with restriction endonuclease EcoRI, which cuts each plasmid once. The digests were run on 0.8% agarose gels in TBE buffer at 90 volts for 90 min and then stained with ethidium bromide for 10 min. DNA band sizes were estimated using a 1Kb DNA ladder (left gel lanes in B and C). Plasmid pcIpR-P-timm is 5292bp, pcIpR-P ^Δ76^-timm is 5064bp, and the high copy pUC19 is 2686bp.

## 4. Conclusions

Our complementation results suggest that only trace levels of *P* expression are needed to catalyze the initiation of λ replication, providing an explanation for early observations about “trans-activation.” It appears that ColE1 replication is extremely sensitive to P, and cells with repressed *P*-encoded plasmids (i.e., with sub-induction levels of *P* expression) can lead to ColE1 plasmid curing. Both P-lethality to cells, and the observed P-dependent cell loss of ColE1 plasmids, were fully suppressed by dual missense mutations altering *dnaB,* or an in frame deletion near the N-terminal end of P. The P-inhibitory phenotypes were partially suppressed by a π missense mutation in *P*, and plasmids expressing the λ encoded OOP antisense micro-RNA. P-dependent cellular filamentation was observed in *ΔrecA* or *lexA*[Ind^-^] cells, considered defective for SOS induction. These studies suggest the hypothesis that cellular levels of P can directly interfere not only with *E. coli* replication initiation, but subsequent steps involving DNA propagation and replication restart. 
